# Research on the Role of Combined Chemotherapy and Radiotherapy in Patients With N+ Non-Metastatic Metaplastic Breast Carcinoma: A Competing Risk Analysis Model Based on the SEER database, 2000 to 2015

**DOI:** 10.3389/fonc.2020.583488

**Published:** 2021-01-22

**Authors:** Yifei Ma, Zejian Yang, Yihan Gao, Kunlong Li, Pei Qiu, Heyan Chen, Shengyu Pu, Bo Wang, Can Zhou

**Affiliations:** ^1^ Department of Breast Surgery, First Affiliated Hospital, Xi’an Jiaotong University, Xi’an, China; ^2^ School of Medicine, Xi’an Jiaotong University, Xi’an, China; ^3^ Department of Computer Science, The University of Hong Kong, Hong Kong, Hong Kong; ^4^ Department of Translational Medicine Center, First Affiliated Hospital of Xi’an Jiaotong University, Xi’an, China

**Keywords:** metaplastic breast carcinoma, combined chemotherapy and radiotherapy, SEER, regional lymph node metastasis, competing risk model

## Abstract

**Purpose:**

Due to the rarity of metaplastic breast carcinoma (MpBC), no randomized trials have investigated the role of combined chemotherapy and radiotherapy (CCRP) in this condition. We aimed to explore and identify the effectiveness of CCRP in patients with regional lymph node metastasis (N+) non-metastatic MpBC.

**Materials and Methods:**

Data were obtained from the National Cancer Institute’s Surveillance, Epidemiology, and End Results (SEER) Program database. We assessed the effects of CCRP on overall survival (OS), breast cancer-specific survival (BCSS), and breast cancer-specific death (BCSD) using Kaplan-Meier analysis, competing risk model analysis, and competing risk regression mode analysis.

**Results:**

A total of 707 women and 361 death cases were included in the unmatched cohort, of which 76.45% (276/361) were BCSD, and 23.55% (85/361) were non-breast cancer-specific deaths (non-BCSD). Both the ChemT and CCRP groups had better OS (ChemT group: HR: 0.59, 95% CI: 0.45–0.78, *P*<0.001; CCRP group: HR: 0.31, 95% CI: 0.23–0.41, *P*<0.001) and BCSS (ChemT group: HR: 0.63, 95% CI: 0.45–0.87, *P*<0.001; CCRP group: HR: 0.32, 95%CI: 0.22–0.46, *P*<0.001) than the non-therapy group. Subjects in the CCRP group tended to have significantly lower cumulative BCSD (Gray’s test, *P*=0.001) and non-BCSD (Gray’s test, *P*<0.001) than the non-therapy group or ChemT group. In competing risk regression model analysis, subjects in the CCRP group had a better prognosis in BCSD (HR: 0.710, 95% CI: 0.508–0.993, *P*=0.045) rather than the ChemT group (HR: 1.081, 95% CI: 0.761–1.535, *P*=0.660) than the non-therapy group.

**Conclusion:**

Our study demonstrated that CCRP could significantly decrease the risk of death for both BCSD and non-BCSD and provided a valid therapeutic strategy for patients with N+ non-metastatic MpBC.

## Introduction

Metaplastic breast carcinoma (MpBC), characterized by the co-existence of carcinoma with non-epithelial cellular elements, is a rare primary breast malignancy. In recent years, MpBC has been mainly classified into seven subtypes: squamous, adenosquamous, spindle cell carcinoma, carcinoma with chondroid or osseous metaplasia, carcinosarcoma, osteoclastic giant cells, and matrix-producing carcinoma subtype (1–3). Previous studies have indicated that patients with MpBC tend to have a poor prognosis and multiple negative features correlated with poor prognosis, such as larger tumors, poorly differentiated grade, and more hormone receptor negativity (HR-) compared with patients with invasive ductal carcinoma [IDC]) (4–8). In addition, treatment for MpBC is relatively unknown due to the rarity of the disease (9). Therefore, more clinical evidence for treatment strategies were needed for MpBC patients since current guidelines were written based on IDC (1, 10).

Multimodality treatment, or combined chemotherapy and radiotherapy (CCRP), is the standard treatment for breast carcinoma due to HR- tumors in patients with MpBC. Most studies have focused on the effects of adjuvant radiotherapy (RT) or chemotherapy (ChemT) alone on long-term outcomes for patients with MpBC and have shown that RT or ChemT alone can improve breast cancer-specific survival (BCSS) and overall survival (OS) in patients with MpBC (1, 11–17). However, few well-performed studies have been conducted on the efficacy of CCRP in patients with resectable MpBC. For these reasons, more studies are urgently needed to confirm the real-world curative effect of CCRP in patients with MpBC, especially those with regional lymph nodes metastasis (N+), who always have a worse prognosis than those with no metastasis of regional lymph nodes (N0) (13).

To further explore and identify the effects of CCRP in patients with regional lymph node metastasis (N+) MpBC, we followed a large cohort of women with N+ MpBC from 2000 to 2015 based on the population-based database Surveillance, Epidemiology, and End Results (SEER) cancer registry program. Statistical methods such as a competing risk analysis model were applied to further investigate the efficiency of CCRP, ChemT, and prognostic factors on MpBC patients with lymph nodes metastasis.

## Materials and Methods

### Data Source

Our study cohort was obtained from the SEER program database, which includes population-based data from 18 cancer registries in about 30% of the U.S. population from 1975 to 2016 (18). It provides complete information, including patient demographics, cancer diagnosis, tumor characteristics, first course of treatment, and follow-up for vital status. In July 2019, we received permission from SEER to analyze the data (SEER ID:14518-Nov2018) with SEER*Stata version 8.3.6. All procedures were performed in accordance with approved guidelines. This study was approved by the Ethics Committee of the First Affiliated Hospital of Xi’an Jiaotong University. Informed patient consent was not required to access and use SEER data.

### Study Population

Women diagnosed with microscopically confirmed MpBC from 2000 to 2015 were enrolled in the study. Patients were included if they met the following criteria: (1) had primary breast cancer (ICD-0-3 primary site codes: C500-C506, C508, and C509); (2) had metaplastic tumor histology (ICD-0-3 morphology codes: 8032, 8035, 8052, 8070-8075, 8560, 8562, 8570-8575, 8980-8982) (18). The following demographic and clinicopathological patient variables were included: age, race, year of diagnosis, marital status, grade, breast-adjusted American Joint Committee on Cancer (AJCC) Sixth tumor-nodes-metastasis (TNM) stage, estrogen receptor (ER) status, progesterone receptor (PR) status, sequence number, surgical procedures, chemotherapy status, radiotherapy status, and cause of death. We only included HER-2 (human epidermal growth factor receptor 2) status in 2010-2015 because SEER database only recorded these data after January 1, 2010 (19).

**Figure 1:**
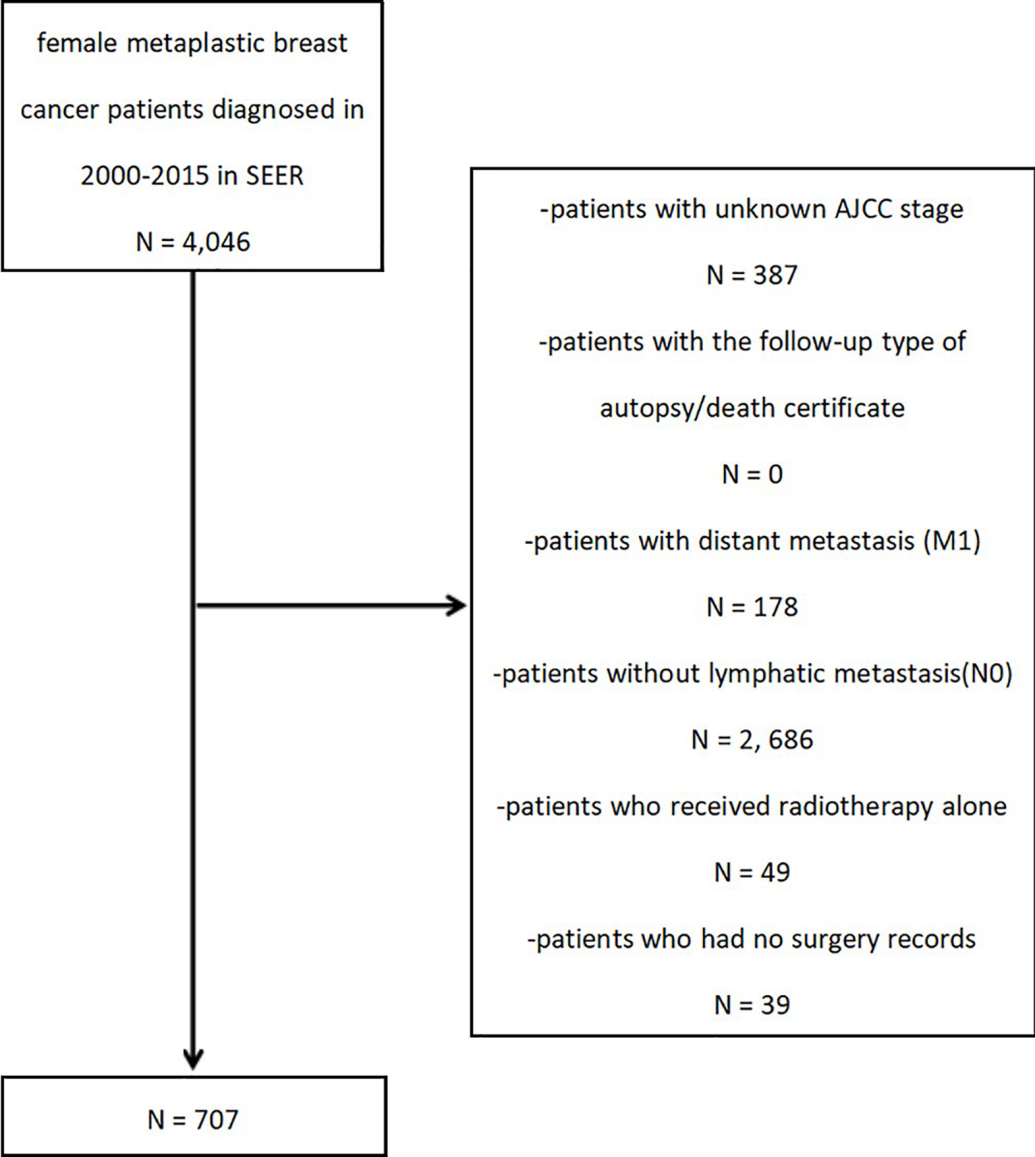
Eligibility, inclusion, and exclusion criteria of study population.

After the preliminary subject selection, patients were excluded if they had the following criteria: (1) unknown AJCC stage; (2) the follow-up type of autopsy or death certificate; (3) distant metastasis (M1); (4) no lymph node metastasis (N0); (5) radiotherapy received alone; or (6) missing surgery records. The selection procedure is shown in **Figure 1**.

A total of 707 patients with MpBC and regional lymph node metastasis (N+) were selected. To evaluate the effect of CCRP on prognosis, the study cohort was classified into one of three groups by treatment method: non-therapy group, ChemT group, and CCRP group.

### End Points

Patients were followed up until November 2018. The primary outcome measurement was overall survival (OS), defined as the time from diagnosis to death, because OS has been used to analyze and compare surgical outcomes among different medical centers. Secondary outcome measurements were breast cancer-specific survival (BCSS) and breast cancer-specific death (BCSD), defined as time from initiation of therapy to living or death from breast carcinoma.

### Statistical Analysis

Multiple statistical methods and models were used to produce an accurate result. The Pearson chi-square test or Fisher’ s exact test were applied to test the independence of patient demographics and treatment-related variables among groups. Kaplan-Meier curve analysis was used to generate OS and BCSS curves, and the log-rank test was used to determine statistical differences among groups. Multivariate Cox regression model was performed to investigate the independent prognostic factors in OS.

A competing risk model analysis was used to mitigate the estimation bias by classifying death causes into two subgroups (20), BCSD and non-BCSD. Gray’s test was used to identify any statistical differences between BCSD and non-BCSD due to any competing risk events. Furthermore, multivariate analysis considering the competing risk events was performed to identify independent prognostic factors in BCSD and non-BCSD, respectively. SPSS version 18.0 (IBM Corporation, Armonk, NY, USA) and R software (version 3.6.1, R Foundation for Statistical Computing, Vienna, Austria. http://www.R-project.org/) were used for our calculations. In addition, we constructed the competing risk model and competing risk regression by R package of cmprsk and foreign. All *P* values were two-sided and *P*<0.05 was considered to be statistically significant (21).

## Results

### Baseline Characteristics of Patients

The baseline clinical characteristics of the included patients are shown in **Table 1**. Of the 707 subjects included from our study cohort, 337 received CCRP, 218 received chemotherapy alone, and 152 received neither CCRP or chemotherapy. Among these women, 71.00% (502) patients were aged 50 or older, 68.32% (483) patients were in N1 status, 71.43% (505) patients were ER negative (ER-), 79.63% (563) patients were PR negative (PR-), and 75.81% (536) patients had only one primary breast cancer. A total of 418 patients (59.12%) were Non-Hispanic white, and 135 (19.09%) patients were Non-Hispanic black. In Total, 329 (46.53%) patients got married, 378 (53.47%) were unmarried or unknown. In terms of diagnosed time, 218 (30.83%) patients were diagnosed between year 2000 and 2005, and 220 (31.12%) patients between year 2006 and 2010, 269 (38.05%) patients between year 2011 and 2015. A total of 77 (10.89%) cases were highly moderately or differentiated (grade I and II), 630 (89.11%) cases were poorly differentiated or undifferentiated (grade III and IV). A total of 317 cases had HER-2 status records between 2010 to 2015, 8.52% (27/317) of them had HER-2 positive (HER-2+) tumors. By comparing CCRP, ChemT and non-therapy groups, significant differences (*P*<0.05) were found in age at diagnosis, marital status, T stage, ER status, PR status, HER-2 status, sequence number and surgical procedures. Key methodological characteristics were shown in **Table 1**.

**Table 1:** Patient clinical and pathological characteristics.

Characteristics	N (707)		Non-therapy (152)		Chemotherapy (218)		CCRP (337)		χ2	p
	N	%	n	%	n	%	n	%		
Age at diagnosis(median)									23.12	<0.001
<50	205	29.00	21	13.82	66	30.28	118	35.01		
≧50	502	71.00	131	86.18	152	69.72	219	64.99		
Race									4.95	0.551
Non-Hispanic White	418	59.12	91	59.87	118	54.13	209	62.02		
Non-Hispanic Black	135	19.09	32	21.05	45	20.64	58	17.21		
Hispanic (All Races)	100	14.14	20	13.16	34	15.60	46	13.65		
Others	54	7.64	9	5.92	21	9.63	24	7.12		
Year of diagnosis									9.16	0.057
2000–2005	218	30.83	51	33.55	65	29.82	102	30.27		
2006–2010	220	31.12	54	35.53	76	34.86	90	26.71		
2011–2015	269	38.05	47	30.92	77	35.32	145	43.03		
Marital status									30.62	<0.001
Married	329	46.53	46	30.26	93	42.66	190	56.38		
Unmarried/Unknown	378	53.47	106	69.74	125	57.34	147	43.62		
Grade									8.41	0.015
I/II	77	10.89	24	15.79	14	6.42	39	11.57		
III/IV	630	89.11	128	84.21	204	93.58	298	88.43		
T stage									6.67	0.353
T1	94	13.30	20	13.16	27	12.39	47	13.95		
T2	335	47.38	71	46.71	117	53.67	147	43.62		
T3	154	21.78	37	24.34	38	17.43	79	23.44		
T4	124	17.54	24	15.79	36	16.51	64	18.99		
N Stage									0.36	0.986
N1	483	68.32	104	68.42	152	69.72	227	67.36		
N2	144	20.37	31	20.39	42	19.27	71	21.07		
N3	80	11.32	17	11.18	24	11.01	39	11.57		
ER Status									4.57	0.102
Negative/Unknown/Borderline	542	76.66	115	75.66	178	81.65	249	73.89		
Positive	165	23.34	37	24.34	40	18.35	88	26.11		
PR Status									5.38	0.068
Negative/Unknown/Borderline	603	85.29	127	83.55	196	89.91	280	83.09		
Positive	104	14.71	25	16.45	22	10.09	57	16.91		
HER-2 Status*									6.33	0.042
Negative/Unknown/Borderline	290	91.48	56	100.00	86	89.58	148	89.70		
Positive	27	8.52	0	0.00	10	10.42	17	10.30		
Sequence number									16.45	0.002
First and only cancer	536	75.81	102	67.11	164	75.23	270	80.12		
First of multiple cancers	67	9.48	13	8.55	22	1.09	32	9.50		
Not first cancer	104	14.71	37	24.34	32	14.68	35	10.39		
Surgery									20.76	<0.001
Mastectomy	548	77.51	127	83.55	185	84.86	236	70.03		
Lumpectomy	159	22.49	25	16.45	33	15.14	101	29.97		

ER, estrogen receptor; PR, progesterone receptor.

HER-2, human epidermal growth factor receptor 2.

BCSD, breast cancer-specific death; non-BCSD, non-breast cancer-specific deaths.

CCRP, combined chemotherapy and radiotherapy.

*The SEER database only recorded HER-2 status after January 1, 2010 (317/707).

### Treatment Methods and Survival Rates

**Figure 2:**
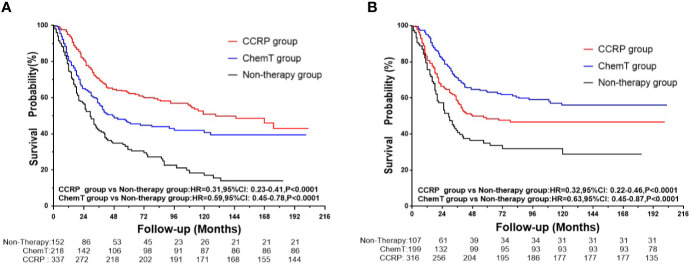
**(A)** OS curves for N+ MpBC patients by treatment methods. **(B)** BCSS curves for N+ MpBC patients stratified by treatment methods.

A total of 361 subjects died (51.06%), with a median follow-up of 36 months (range, zero to 203 months). The cumulative OS at 5, 10, and 15 years from all causes was 31.34, 16.95, and 13.98% in the non-therapy group, respectively; 46.18, 40.66, and 39.25% in the ChemT group, respectively; and 62.14, 50.84, and 42.86% in the CCRP group, respectively (**Figure 2A**). The hazard ratio (HR) summarized the risk of OS and BCSS. As shown in **Figure 2A**, the HRs of 0.59 (95% CI: 0.45–0.78, *P*<0.001) in the ChemT group and 0.31 (95% CI: 0.23–0.41, *P*<0.001) in the CCRP group indicated that ChemT and CCRP could confer an OS advantage for patients with N+ MpBC, with the risk of death from all causes reduced by 41 and 69%, respectively.

As shown in **Figure 2B**, the cumulative BCSS at 5, 10, and 15 years was 35.02, 28.90, and 28.90% in the non-therapy group, respectively; 48.45, 46.67, and 46.67% in the ChemT group, respectively; and 63.02, 55.96, and 55.96% in the CCRP group, respectively. The HRs of 0.63 (95% CI: 0.45–0.87, *P*<0.001) and 0.32 (95% CI: 0.22–0.46, *P*<0.001) demonstrated that the risk of BCSD could be lowered by 37 and 68% through ChemT and CCRP, respectively, for patients with N+ MpBC.

### Competing Risk Model Analysis of BCSD and Non-BCSD

**Figure 3:**
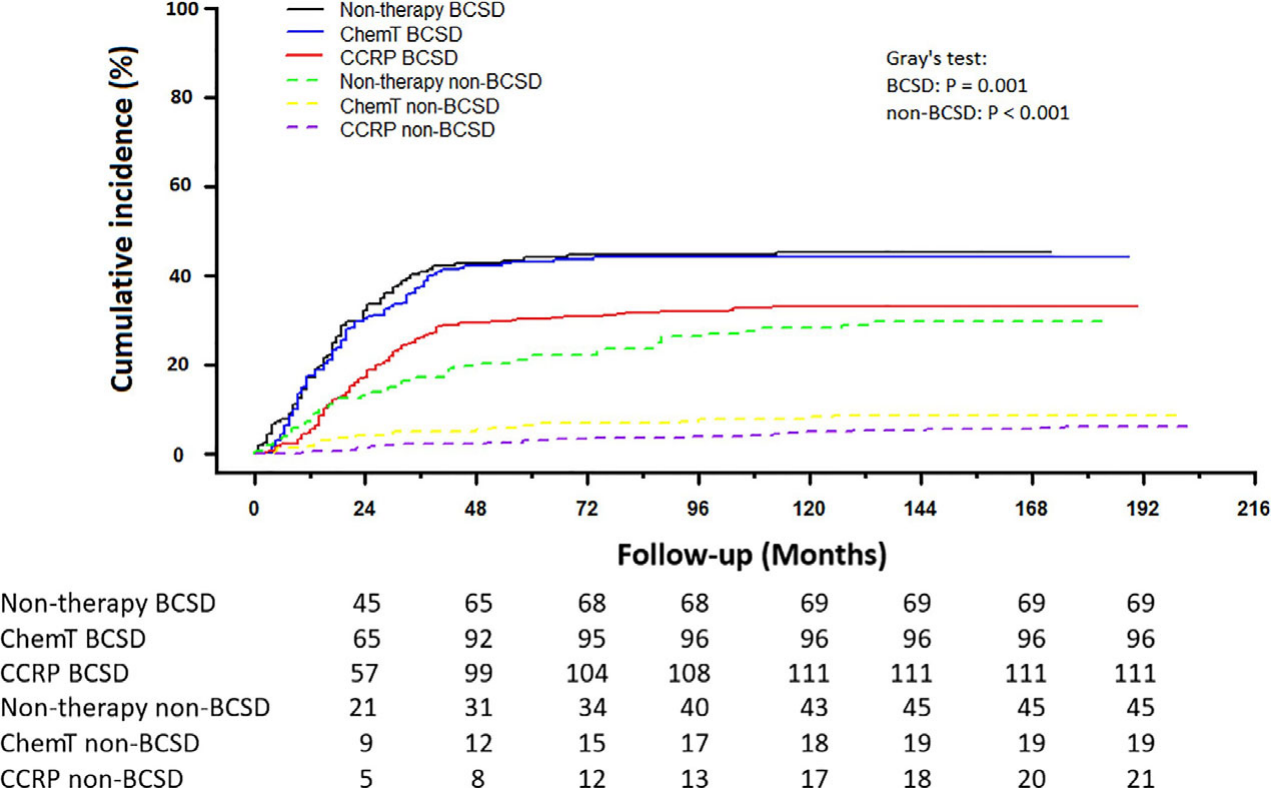
Cumulative incidence of BCSD and non-BCSD in N+ MpBC patients by treatment methods.

Of the 361 deaths out of 707 patients, the total cumulative incidence of BCSD was 39.04% (276), with the non-BCSD rate as high as 12.02% (85). As shown in **Figure 3**, patients in the CCRP group had better cumulative BCSD incidence (Gray’s test, *P*=0.001) and non-BCSD incidence (Gray’s test, *P*<0.001) than did the non-therapy group and ChemT group. Moreover, compared with the non-therapy group, patients in the ChemT group tended to have lower cumulative non-BCSD incidence than BCSD incidence. In addition, mortality ratios for all causes of death were calculated in **Table S1** and **Figure S1**. In total, 76.45% (276/361) participants died from breast cancer and 3.60% (13/361) died from other cancers (non-breast). 19.94% (72/361) cases died from non-neoplastic diseases such as cardiovascular and cerebrovascular diseases (7.76%, 28/361), respiratory diseases (3.88%, 14/361) and genitourinary disease (2.22%, 8/361). Moreover, 94.93% (262/276) of the BCSD occurred within 5 years of diagnosis.

### Multivariate Cox Regression Model Analysis

The independent prognostic factors in OS were analyzed using a multivariate Cox regression model (**Table 2**). Patients in both CCRP group and ChemT group had better OS than non-therapy group (ChemT: HR: 0.648, 95% CI: 0.494–0.850, *P*=0.002; CCRP: HR: 0.396, 95% CI: 0.302–0.518, *P*<0.001). Patients in the lumpectomy subgroup showed a better OS than those in the mastectomy subgroup (HR: 0.645, 95% CI: 0.459–0.906, *P*=0.012). Patients in T3 subgroup and T4 stage subgroup had worse OS than those in T1 stage subgroup (T3 stage: HR: 2.196, 95% CI: 1.441–3.346, *P<*0.001; T4 stage: HR: 2.719, 95% CI: 1.776–4.186, *P*<0.001). The HRs 1.572 (95% CI: 1.223–2.019, *P*<0.001) and 1.904 (95% CI: 1.403–2.586, *P*<0.001) indicated that patients in N2 subgroup and N3 stage subgroup had worse OS, than those in the N1 subgroup, respectively.

**Table 2:** Multivariate COX proportional risk model.

Characteristics	Hazard ratio	95% CI	P value
Age at diagnosis(median)			
<50	1	—	—
≧50	1.251	0.969–1.614	0.085
Race			
Non-Hispanic White	1	—	—
Non-Hispanic Black	0.899	0.683–1.183	0.448
Hispanic (All Races)	0.725	0.516–1.020	0.065
Other races	0.792	0.515–1.218	0.289
Year of diagnosis			
2000-2005	1	—	—
2006-2010	1.107	0.858–1.429	0.435
2011-2015	1.044	0.783–1.391	0.769
Marital status			
Married	1	—	—
Unmarried/Unknown	1.101	0.876–1.384	0.411
Grade			
I/II	1	—	—
III/IV	1.299	0.900–1.876	0.163
T stage			
T1	1	—	—
T2	1.139	0.768–1.690	0.518
T3	2.196	1.441–3.346	<0.001
T4	2.719	1.766–4.186	<0.001
N Stage			
N1	1	—	—
N2	1.572	1.223–2.019	<0.001
N3	1.904	1.403–2.586	<0.001
ER Status			
Negative/Unknown/Borderline	1	—	—
Positive	0.718	0.511–1.010	0.057
PR Status			
Negative/Unknown/Borderline	1	—	—
Positive	1.013	0.684–1.500	0.951
Sequence number			
First and only cancer	1	—	—
First of multiple cancers	0.870	0.612–1.237	0.438
Not first cancer	1.211	0.907–1.618	0.194
Surgery			
Mastectomy	1	—	—
Lumpectomy	0.645	0.459–0.906	0.012
Treatment			
Non-therapy	1	—	—
ChemT	0.648	0.494–0.850	0.002
CCRP	0.396	0.302–0.518	<0.001

ER, estrogen receptor; PR, progesterone receptor.

BCSD, breast cancer-specific death; non-BCSD, non-breast cancer-specific deaths.

CCRP, combined chemotherapy and radiotherapy.

### Competing Risk Regression Model Analysis

To further investigate the independent prognostic factors in BCSD, a competing risk regression model analysis was performed (**Table 3**). Unexpectedly, the CCRP group had better BCSD (HR: 0.710, 95% CI: 0.508–0.993, *P*=0.045) and non-BCSD (HR: 0.258, 95% CI: 0.148–0.449, *P*<0.001) than the non-therapy and ChemT groups. However, the ChemT group had no better BCSD than the non-therapy group (HR: 1.081, 95% CI: 0.761–1.535, *P*=0.660). The lumpectomy subgroup had better BCSD than the mastectomy subgroup, respectively (HR: 0.639, 95% CI: 0.433–0.942, *P*=0.024). The HRs 2.476 (95% CI: 1.504–4.077, *P*<0.001) and 3.504 (95% CI: 2.106–5.830, *P*<0.001) indicated that patients in the two subgroups had a worse BCSD in T3 stage subgroup and T4 stage subgroup, respectively, than that in the T1 stage subgroup. The N1 stage subgroup showed a better BCSD than the N2 subgroup (HR: 1.619, 95% CI: 1.199–2.186, *P=*0.002) and N3 subgroup (HR: 1.766, 95% CI: 1.258–2.478, *P=*0.001). The ChemT subgroup had better non-BCSD (HR: 0.318, 95% CI: 0.182–0.555, *P*<0.001) than the non-therapy subgroup. In addition, both the first and not first of the multiple-cancer subgroups had worse non-BCSD (HR: 2.674, 95% CI: 1.490–4.796, *P*=0.001; HR: 1.998, 95% CI: 1.131–3.531, *P*=0.017) than that in the first and only single-cancer subgroup. Moreover, to investigate the prognostic value of HER-2 status, we also performed an analysis for patients diagnosed after 2010. As shown in **Table S2**, HER-2 status was not associated with BCSD and had no effects on patients in CCRP subgroup, ChemT subgroup or non-therapy subgroup.

**Table 3:** Multivariate COX proportional risk models considering competitive risk.

Characteristics	BCSD (N1 = 276,76.45%)			non-BCSD (N2 = 85, 23.55%)		
	Hazard ratio	95% CI	P value	Hazard ratio	95% CI	P value
Age at diagnosis(median)						
<50	1	—	—	1	—	—
≧50	1.096	0.824–1.458	0.530	1.955	0.981–3.895	0.057
Race						
Non-Hispanic White	1	—	—	1	—	—
Non-Hispanic Black	1.096	0.796–1.509	0.570	0.662	0.353–1.242	0.200
Hispanic (All Races)	0.740	0.510–1.073	0.110	0.887	0.450–1.748	0.730
Other races	0.819	0.473–1.420	0.480	0.949	0.367–2.457	0.910
Year of diagnosis						
2000–2005	1	—	—	1	—	—
2006–2010	0.933	0.697–1.248	0.640	1.245	0.738–2.098	0.410
2011–2015	0.721	0.523–0.995	0.046	1.205	0.677–2.145	0.530
Marital status						
Married	1	—	—	1	—	—
Unmarried/Unknown	0.957	0.734–1.247	0.750	1.486	0.904–2.444	0.120
Grade						
I/II	1	—	—	1	—	—
III/IV	1.214	0.824–1.787	0.330	1.261	0.609–2.613	0.530
T stage						
T1	1	—	—	1	—	—
T2	1.442	0.904–2.299	0.120	0.706	0.365–1.366	0.300
T3	2.476	1.504–4.077	<0.001	0.980	0.464–2.066	0.960
T4	3.504	2.106–5.830	<0.001	0.694	0.317–1.521	0.360
N Stage						
N1	1	—	—	1	—	—
N2	1.619	1.199–2.186	0.002	0.919	0.511–1.652	0.780
N3	1.766	1.258–2.478	0.001	1.233	0.588–2.588	0.580
ER Status						
Negative/Unknown/Borderline	1	—	—	1	—	—
Positive	0.675	0.455–1.001	0.051	1.197	0.626–2.291	0.590
PR Status						
Negative/Unknown/Borderline	1	—	—	1	—	—
Positive	1.042	0.676–1.604	0.850	0.977	0.447–2.132	0.950
Sequence number						
First and only cancer	1	—	—	1	—	—
First of multiple cancers	0.644	0.412–1.007	0.054	2.674	1.490–4.796	0.001
Not first cancer	0.960	0.665 1.387	0.830	1.998	1.131–3.531	0.017
Surgery						
Mastectomy	1	—	—	1	—	—
Lumpectomy	0.639	0.433–0.942	0.024	0.681	0.332–1.398	0.300
Treatment						
Non-therapy	1	—	—	1	—	—
ChemT	1.081	0.761–1.535	0.660	0.318	0.182–0.555	<0.001
CCRP	0.710	0.508–0.993	0.045	0.258	0.148–0.449	<0.001

ER, estrogen receptor; PR, progesterone receptor.

BCSD, breast cancer-specific death; non-BCSD, non-breast cancer-specific deaths.

CCRP, combined chemotherapy and radiotherapy.

### Surgical Procedures and Survival for Patients With N+ MpBC

**Figure 4:**
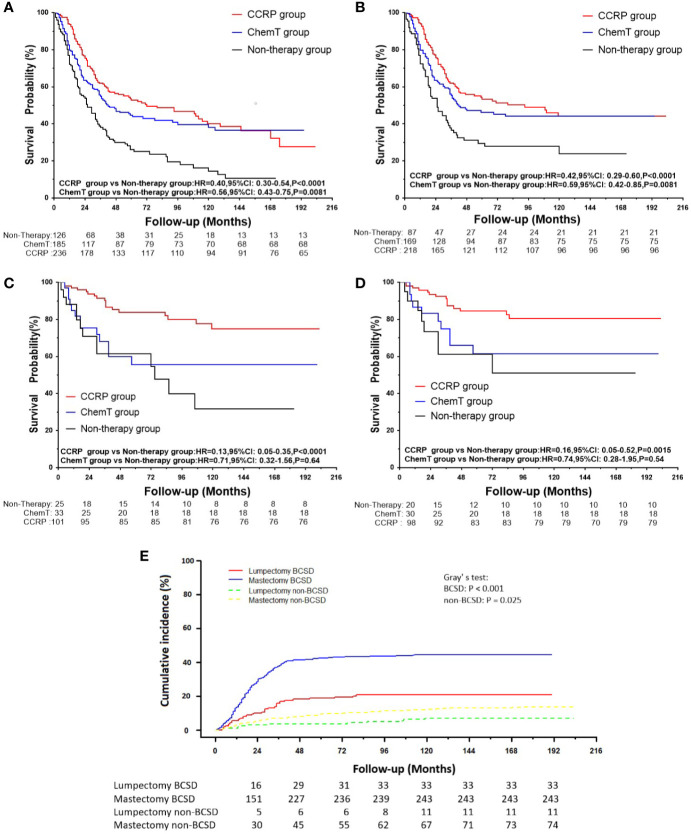
**(A)** OS curves for patients with N+ MpBC undergoing mastectomy. **(B)** BCSS curves for patients with N+ MpBC undergoing mastectomy; **(C)** OS for patients with N+ MpBC undergoing lumpectomy; **(D)** BCSS curves for patients with N+ MpBC undergoing lumpectomy; **(E)** Cumulative incidence of BCSD and non-BCSD by surgical procedures.

A total of 548 patients underwent mastectomy, and 159 underwent lumpectomy (**Table 1**). As shown in **Figures 4A, B**, after mastectomy, patients with N+ MpBC in the CCRP and ChemT groups tended to have higher OS and BCSS than those in the non-therapy subgroup, with the HRs of OS at 0.40 (95% CI: 0.30–0.54, *P*<0.001) and 0.56 (95% CI: 0.43–0.75, *P*=0.0081) and BCSS at 0.42 (95% CI: 0.29–0.60, *P*<0.001) and 0.59 (95% CI: 0.42–0.85, *P*=0.0081) in the CCRP and ChemT group, respectively. Nevertheless, for patients underwent lumpectomy (**Figures 4C, D**), only the CCRP group had higher OS and BCSS, with the HRs of 0.13 in OS (95% CI: 0.050–0.350, *P<*0.0001) and 0.16 in BCSS (95% CI: 0.050–0.520, *P*=0.0015). After competing risk model analysis (**Figure 4E**), patients who underwent lumpectomy in the CCRP group tended to have both lower cumulative BCSD (Gray’s test, *P*<0.001) and non-BCSD (Gray’s test, *P*=0.025) than the other two groups.

### Primary Breast Cancer Sequences and Survival for Patients With N+ MpBC

**Figure 5:**
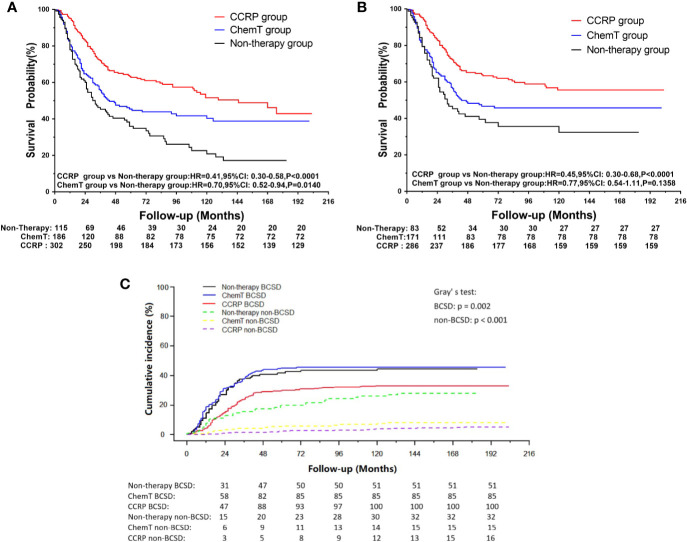
**(A)** OS curves for N+ MpBC patients in PBC subgroup by treatment methods. **(B)** BCSS curves for N+ MpBC patients in PBC subgroup stratified by treatment methods; **(C)** Cumulative incidence of BCSD and non-BCSD in PBC subgroup of N+ MpBC patients by treatment methods.

To further explore the predictive consequences of primary breast carcinoma sequences in multiple primary carcinomas on OS and BCSS, and BCSD, a subgroup analysis was performed (**Table 4**, **Figures 5A–C**). In all, 603 eligible patients were classified into primary breast cancer (PBC) subgroup, with a median follow‐up of 38 months (range, zero to 203 months) and 104 cases into second primary breast cancer (SPBC) subgroup, with a median follow-up of 28.5 months (range, 1 to 177 months). After competing risk regression model analysis, it was discovered that, while deemed as a significant independent prognostic factor on BCSD in SPBC group, CCRP was no longer a significantly independent prognostic factor on BCSD in the PBC subgroup (**Table 5**). As shown in **Figures 5A, B**, patients with PBC in the CCRP tended to have higher OS and BCSS than those in the non-therapy subgroup, although the value of HR was 0.832 (95% CI: 0.573–1.208, *P*=0.330>0.05). Moreover, patients in the CCRP group tended to have both lower cumulative incidence of BCSD (Gray’s test, *P*=0.002) and non-BCSD (Gray’s test, *P*<0.001) than those in the other two groups in the PBC subgroup (**Figure 5C**).

**Table 4:** Multivariate COX proportional risk models considering competitive risk in PBC subgroup.

Characteristics	BCSD (N1 = 236,78.93%)			non-BCSD (N2 = 63, 21.07%)		
	Hazard ratio	95% CI	P value	Hazard ratio	95% CI	P value
Age at diagnosis(median)						
<50	1	—	—	1	—	—
≧50	1.168	0.859–1.589	0.320	2.134	0.984–4.629	0.055
Race						
Non-Hispanic White	1	—	—	1	—	—
Non-Hispanic Black	1.137	0.809–1.599	0.460	0.663	0.307–1.433	0.300
Hispanic (All Races)	0.635	0.421–0.958	0.030	0.986	0.424–2.292	0.970
Other races	0.814	0.456–1.451	0.480	1.033	0.378–2.824	0.950
Year of diagnosis						
2000–2005	1	—	—	1	—	—
2006–2010	0.940	0.685–1.288	0.700	1.199	0.663–2.167	0.550
2011–2015	0.736	0.518–1.045	0.087	1.168	0.595–2.293	0.650
Marital status						
Married	1	—	—	1	—	—
Unmarried/Unknown	1.028	0.771–1.371	0.850	1.122	0.635–1.983	0.690
Grade						
I/II	1	—	—	1	—	—
III/IV	1.373	0.896–2.105	0.150	1.320	0.603–2.893	0.490
T stage						
T1	1	—	—	1	—	—
T2	1.324	0.786–2.231	0.290	1.023	0.442–2.366	0.960
T3	2.501	1.435–4.357	0.001	1.228	0.483–3.125	0.670
T4	3.409	1.937–6.001	<0.001	0.873	0.314–2.426	0.790
N Stage						
N1	1	—	—	1	—	—
N2	1.426	1.020–1.992	0.038	1.240	0.627–2.450	0.540
N3	1.726	1.196–2.489	0.004	1.273	0.548–2.960	0.570
ER Status						
Negative/Unknown/Borderline	1	—	—	1	—	—
Positive	0.785	0.524–1.177	0.240	0.728	0.307–1.725	0.470
PR Status						
Negative/Unknown/Borderline	1	—	—	1	—	—
Positive	0.955	0.597–1.527	0.850	1.308	0.513–3.334	0.570
Sequence number						
First and only cancer	1	—	—	1	—	—
First of multiple cancers	0.656	0.417–1.030	0.067	2.575	1.409–4.709	0.002
Surgery						
Mastectomy	1	—	—	1	—	—
Lumpectomy	0.566	0.367–0.874	0.010	0.616	0.275–1.383	0.240
Treatment						
Non-therapy	1	—	—	1	—	—
ChemT	1.251	0.845–1.852	0.260	0.272	0.146–0.507	<0.001
CCRP	0.832	0.573–1.208	0.330	0.213	0.111–0.408	<0.001

ER, estrogen receptor; PR, progesterone receptor.

BCSD, breast cancer-specific death; non-BCSD, non-breast cancer-specific deaths.

CCRP, combined chemotherapy and radiotherapy.

**Table 5:** Multivariate COX proportional risk models considering competitive risk of BCSD in SPBC subgroup.

Characteristics	BCSD (N=40, 64.52%)		
	Hazard ratio	95% CI	P value
Age at diagnosis(median)			
<50	1	—	—
≧50	0.855	0.350–2.092	0.730
Race			
Non-Hispanic White	1	—	—
Non-Hispanic Black	1.076	0.381–3.036	0.890
Hispanic (All Races)	1.383	0.475–4.028	0.550
Other races	1.392	0.248–7.810	0.710
Year of diagnosis			
2000–2005	1	—	—
2006–2010	0.917	0.366–2.295	0.850
2011–2015	0.504	0.173–1.464	0.210
Marital status			
Married	1	—	—
Unmarried/Unknown	0.467	0.196–1.117	0.087
Grade			
I/II	1	—	—
III/IV	0.839	0.271–2.596	0.760
T stage			
T1	1	—	—
T2	2.522	0.936–6.793	0.067
T3	4.298	1.240–14.898	0.022
T4	3.420	1.050–11.128	0.041
N Stage			
N1	1	—	—
N2	3.009	1.320–6.861	0.009
N3	2.398	0.662–8.680	0.180
ER Status			
Negative/Unknown/Borderline	1	—	—
Positive	0.450	0.140–1.445	0.180
PR Status			
Negative/Unknown/Borderline	1	—	—
Positive	0.905	0.327–2.504	0.850
Surgery			
Mastectomy	1	—	—
Lumpectomy	1.096	0.407–2.954	0.860
Treatment			
Non-therapy	1	—	—
ChemT	0.588	0.237–1.461	0.250
CCRP	0.405	0.167–0.982	0.046

ER, estrogen receptor; PR, progesterone receptor.

BCSD, breast cancer-specific death; non-BCSD, non-breast cancer-specific deaths.

CCRP, combined chemotherapy and radiotherapy.

## Discussion

In this study, we confirmed that the CCRP group had a better prognosis than the ChemT group alone in women with MpBC and lymph nodes metastasis (N+). Based on analysis of a large cohort of 707 subjects in the SEER database from 2000 to 2015 and using an integrated range of factors into a competing risk regression model, the use of CCRP could confer the advantage of improved OS and BCSS by reducing 69% of the risk of death from all causes and 68% of the risk of BCSD for patients with N+ MpBC. To our knowledge, this was the first and largest population-based study to assess the impact of CCRP on patients with MpBC by analyzing survival variables and demographic and pathological factors.

Clinicopathological features such as age, tumor grade, TNM stage, and ER status have always been seemed objective and reliable prognostic indicators that could guide clinical therapy in patients with breast carcinoma. In our study, of the total 707 N+ MpBC participants, 71.43% (505) were ER negative, and 79.63% (563) were PR negative. These findings are similar to those of previous trials that showed that MpBC is associated with older age and fewer ER-positive tumors (2, 4, 5, 22). Therefore, our findings confirm that patients with N+ MpBC have multiple negative features related to poor outcomes.

OS and BCSS are both objective, reliable, precise, and bias-free measurements for patients with breast carcinoma. In our study, 47.67% (337) patients underwent CCRP, 30.83% (218) received chemotherapy alone, and 21.50% (152) received neither chemotherapy nor radiotherapy. After Kaplan-Meier curve analysis, both CCRP and ChemT groups had improved OS and BCSS compared with the non-therapy group. Undoubtedly, such results indicate that CCRP and ChemT could significantly prolong OS and BCSS in patients with N+ MpBC. However, the estimation bias resulting from other causes of death (non-BCSD), which might be a competing risk affecting BCSD and preclude the occurrence of the primary event, should not be ignored.

To mitigate the estimation bias and further investigate the efficiency of CCRP and ChemT on BCSD or non-BCSD for patients with MpBC, a competing risk regression model analysis was used (20, 21, 23, 24). We found that CCRP, not ChemT, could significantly decrease the risk of BCSD and non-BCSD for patients with N+ MpBC. Therefore, the administration of CCRP plays a vital role in decreasing BCSD and non-BCSD and should be a treatment strategy for patients with N+ MpBC.

The administration of CCRP may eradicate residual microtumors and improve the control of micro-metastatic foci and then reduce the risk of recurrence in the ipsilateral breast and long-distance metastasis, as previously described (13, 14, 25–30). Our findings are consistent with those of previous studies in that MpBC patients could benefit from removal of the tumor and adjuvant radiation therapy and systemic chemotherapy (2, 9). Even systemic chemotherapy for patients with MpBC is thought to be suboptimal compared with standard IDC (2, 9, 22) due to reduced or loss of ER and PR receptor expression and insensitivity to chemotherapy compared with IDC patients, as previous small studies have indicated (13, 14, 26–30).

To investigate the effect of surgical procedures on survival due to the favorable BCSD results from the competing risk model analysis in the lumpectomy subgroup through the administration of CCRP, a subgroup analysis was applied. In the Kaplan-Meier curve analysis, significant increases were observed in respect to OS and BCSS in the lumpectomy subgroup for patients with N+ MpBC through the administration of CCRP rather than ChemT compared with the mastectomy subgroup. Theoretically, the introduction of CCRP after lumpectomy may treat the microscopic tumor foci, which may remain in the conserved breast after removal of the macroscopic and detected focus and lead to locoregional recurrence or life-threatening distant metastases and reduce the risk of recurrence in the ipsilateral breast (25, 31–33). In addition, the small population size of 20 to 33 participants in the non-therapy and ChemT subgroups may have greatly influenced the occurrence of the primary event of interest.

Due to the high incidence and the long life expectancy, patients with breast carcinoma showed higher incidence of second primary breast cancer (SPBC) and therefore was associated with poorer prognosis (34). To further explore the predictive consequences of primary breast cancer (PBC) on OS, BCSS and BCSD, a subgroup analysis was performed. Through Kaplan-Meier curve analysis, significant increases in OS and BCSS were observed in patients with N+ MpBC through the administration of CCRP. However, while deemed as a significant independent prognostic factor on BCSD in SPBC group, it appears that CCRP was no longer a significantly independent prognostic factor on BCSD in the PBC subgroup. The underlying reason may be the good prognosis of MpBC and insufficient follow-up time. The value of HR 0.832 (0.573–1.208), indicated that the administration of CCRP tended to decrease the risk of BCSD after adequate follow-up time.

This study was not without limitations. Selection bias may have occurred due to the nature of the retrospective analysis. Studies that randomly assign patients into different groups by treatment method are desperately needed. An additional limitation of the current study was the lack of information about detailed regimens and sequence of chemotherapy, endocrine therapy, radiation dose, and targeted therapy against HER-2/neu-overexpression or clinical target volume. Records regarding the patterns of locoregional and distant recurrence after treatment were also unavailable. Moreover, more detailed information about patients, such as frailty or co-morbid conditions known to be related to receipt of specific treatments, was also unavailable. Finally, and most importantly, median follow-up time in this study was 36 months. Longer follow-up time is necessary for an accurate assessment of prognostic factors for patients with N+ MpBC. However, we believe that the findings of this study, which cover about 28% of the U.S. population of patients with cancer, are generalizable and will contribute to improved survival in N+ non-metastatic MpBC patients.

## Conclusion

In conclusion, our research demonstrated that CCRP, but not chemotherapy alone, can significantly decrease the risk of BCSD for patients with N+ MpBC. In view of our study’s results, we conclude that MpBC is associated with poor prognostic variables and that CCRP could be an effective treatment strategy for patients with N+ MpBC. Randomized controlled clinical trials with long follow-up time are still needed to provide a high level of evidence on advantages of CCPR for patients with N+ non-metastatic metaplastic PBC patients.

## Data Availability Statement

The datasets presented in this study can be found in online repositories. The names of the repository/repositories and accession number(s) can be found below: https://seer.cancer.gov/, Surveillance, Epidemiology, and End Results (SEER) cancer registry program, SEER ID:14518-Nov2018.

## Ethics Statement

The studies involving human participants were reviewed and approved by Ethics Committee of the First Affiliated Hospital of Xi’an Jiaotong University. Written informed consent for participation was not required for this study in accordance with the national legislation and the institutional requirements.

## Author Contributions

Study concept and design: CZ and BW. Acquisition, analysis, or interpretation of data: all authors. Drafting of the manuscript: YM, CZ, ZY, and PQ. Critical revision of manuscript: CZ, BW, and SP. Statistical analysis: YM, YG, KL, PQ, and HC. Obtained funding: CZ and BW. Administrative, technical, and material support: YG. Study supervision: CZ and BW. All authors contributed to the article and approved the submitted version.

## Funding

This work was supported by the National Natural Science Foundation of China (Grant No: 81502413) and Shaanxi Provincial Natural Science Foundation of China (Grant NO.2019SF-145).

## Conflict of Interest

The authors declare that the research was conducted in the absence of any commercial or financial relationships that could be construed as a potential conflict of interest.

## Supplementary Material

Supplementary Figure 1 Causes of death in each latency period following N+ MpBC diagnosis are illustrated.

Supplementary Table 1 Causes of death for N+ MpBC patients in each latency period after diagnosed.

Supplementary Table 2 Multivariate COX proportional risk models considering competitive risk of BCSD in patients diagnosed 2010+ (with HER-2 status). ER, estrogen receptor; PR, progesterone receptor. HER-2, human epidermal growth factor receptor 2. BCSD, breast cancer-specific death; non-BCSD, non-breast cancer-specific deaths. CCRP, combined chemotherapy and radiotherapy.
