# Supplementary figures and images for: Research on the Role of Combined Chemotherapy and Radiotherapy in Patients With N+ Non-Metastatic Metaplastic Breast Carcinoma: A Competing Risk Analysis Model Based on the SEER database, 2000 to 2015
Source: Front Oncol. 2021 Jan 22;10:583488. doi: 10.3389/fonc.2020.583488 (PMC7862760; doi:10.3389/fonc.2020.583488)

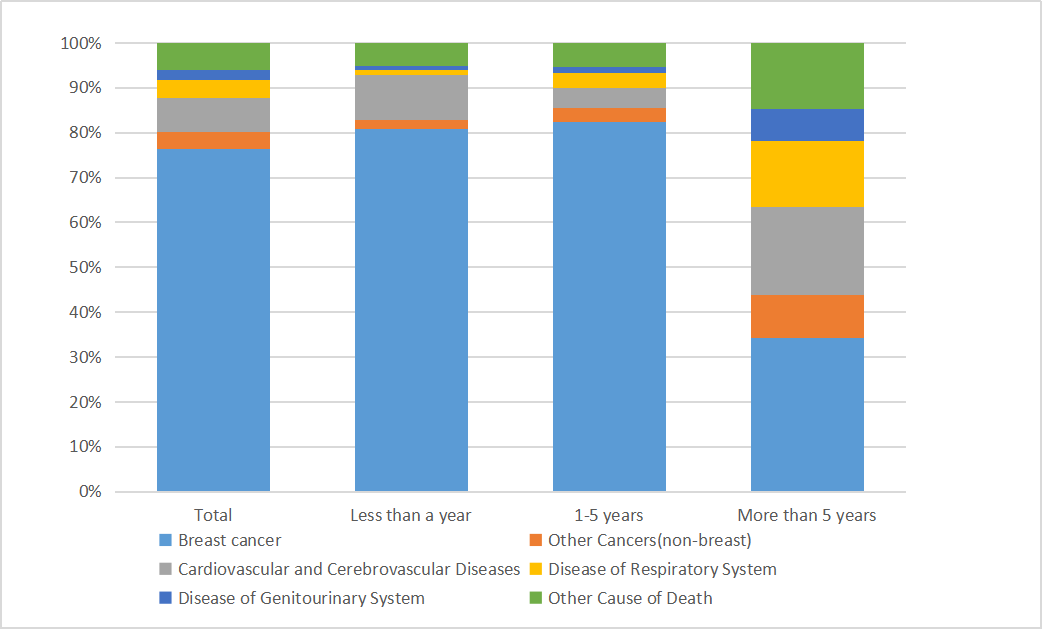

Supplement: Supplementary Figure 1 — Causes of death in each latency period following N+ MpBC diagnosis are illustrated. [file Image_1.png]
