# Supplementary material for: Research on the Role of Combined Chemotherapy and Radiotherapy in Patients With N+ Non-Metastatic Metaplastic Breast Carcinoma: A Competing Risk Analysis Model Based on the SEER database, 2000 to 2015
Source: Front Oncol. 2021 Jan 22;10:583488. doi: 10.3389/fonc.2020.583488 (PMC7862760; doi:10.3389/fonc.2020.583488)
Supplement: Supplementary Table 1 — Causes of death for N+ MpBC patients in each latency period after diagnosed. [file DataSheet_1.doc]

TABLE S1 Causes of death for N+ MpBC patients in each latency period after diagnosed

| Diseases | **Total(361)** | | **< 1 year(99)** | | **1-5 years(221)** | | **> 5 years(41)** | |
| --- | --- | --- | --- | --- | --- | --- | --- | --- |
|  | N | % | N | % | N | % | N | % |
| Breast cancer | 276 | 76.45 | 80 | 80.81 | 182 | 82.35 | 14 | 34.15 |
| Other Cancers (non-breast) | 13 | 3.60 | 2 | 2.02 | 7 | 3.17 | 4 | 9.76 |
| Cardiovascular and Cerebrovascular Diseases | 28 | 7.76 | 10 | 10.10 | 10 | 4.52 | 8 | 19.51 |
| Disease of Respiratory System | 14 | 3.88 | 1 | 1.01 | 7 | 3.17 | 6 | 14.63 |
| Disease of Genitourinary System | 8 | 2.22 | 1 | 1.01 | 3 | 1.36 | 3 | 7.32 |
| Other Causes of Death | 22 | 6.09 | 5 | 5.05 | 12 | 5.43 | 6 | 14.63 |

TABLE S2 |Multivariate COX proportional risk models considering competitive risk of BCSD in patients diagnosed 2010+ (with HER-2 status)

| Characteristics | **BCSD**（N=93， 76.23%） | | |
| --- | --- | --- | --- |
|  | Hazard ratio | 95% CI | P value |
| Age at diagnosis(median) |  |  |  |
| ＜50 | 1 | — | — |
| ≧50 | 1.249 | 0.703-2.219 | 0.450 |
| Race |  |  |  |
| Non-Hispanic White | 1 | — | — |
| Non-Hispanic Black | 1.083 | 0.587-1.998 | 0.800 |
| Hispanic (All Races) | 1.759 | 0.886-3.495 | 0.110 |
| Other races | 0.636 | 0.325-1.246 | 0.190 |
| Marital status |  |  |  |
| Married | 1 | — | — |
| Unmarried/Unknown | 0.992 | 0.619-1.589 | 0.970 |
| Grade |  |  |  |
| I/II | 1 | — | — |
| III/IV | 0.580 | 0.238-1.410 | 0.230 |
| T stage |  |  |  |
| T1 | 1 | — | — |
| T2 | 1.859 | 0.674-5.131 | 0.230 |
| T3 | 4.308 | 1.465-12.670 | 0.008 |
| T4 | 5.818 | 1.861-18.192 | 0.003 |
| N Stage |  |  |  |
| N1 | 1 | — | — |
| N2 | 1.759 | 1.054-2.935 | 0.031 |
| N3 | 0.995 | 0.449-2.202 | 0.990 |
| ER Status |  |  |  |
| Negative/Unknown/Borderline | 1 | — | — |
| Positive | 0.721 | 0.421-1.235 | 0.230 |
| PR Status |  |  |  |
| Negative/Unknown/Borderline | 1 | — | — |
| Positive | 1.261 | 0.608-2.612 | 0.530 |
| HER-2 Status |  |  |  |
| Negative/Unknown/Borderline | 1 | — | — |
| Positive | 0.643 | 0.282-1.464 | 0.290 |
| Surgery |  |  |  |
| Mastectomy | 1 | — | — |
| Lumpectomy | 0.825 | 0.421-1.618 | 0.580 |
| Treatment |  |  |  |
| Non-therapy | 1 | — | — |
| ChemT | 1.097 | 0.578-2.083 | 0.780 |
| CCRP | 0.455 | 0.246-0.845 | 0.013 |

ER: estrogen receptor; PR: progesterone receptor;

HER-2: human epidermal growth factor receptor 2;

BCSD: breast cancer-specific death; non-BCSD: non-breast cancer-specific deaths;

CCRP: combined chemotherapy and radiotherapy.
